# Nursing Professionals within the Intergenerational Context during the 20^th^ and 21^st^ Centuries: an Integrative Review

**DOI:** 10.17533/udea.iee.v39n3e14

**Published:** 2021-11-08

**Authors:** Susana Rollan Oliveira, José Siles González

**Affiliations:** 1 Nurse, PhD candidate. Universidad de Alicante, Spain. Email: rollansusana@hotmail.com Universidad de Alicante Universidad de Alicante Spain rollansusana@hotmail.com; 2 Nurse, PhD. Professor. Universidad de Alicante, Spain. Email: jose.siles@ua.es Universidad de Alicante Universidad de Alicante Spain jose.siles@ua.es

**Keywords:** veterans, intergeneration interval, nurses, delivery of health care., veteranos, brecha generacional, enfermeras y enfermeros, atención a la salud., veteranos, intervalo entre gerações, enfermeiras e enfermeiros, atenção à saúde.

## Abstract

**Objective:**

To describe the generational differences and similarities existing among nursing professionals of the 20th and 21st centuries and how these have influenced on the evolution of the profession.

**Methods:**

Integrative review according to the methodology by Whittemore and Knafl. The key words used for the search were: nurses, intergenerational relations, Veterans, Baby Boom, X generation, and Millennials.

**Results:**

The electronic search process yielded 10 documents (eight articles and two theses), all within the Anglo-Saxon environment (4 in Canada, 5 in the United States, and 1 in Australia). The documents recovered determined three principal themes: *the intergenerational nursing workforce (n =* 7), *recruiting and retention within an intergenerational workforce (n =* 2), and *tutoring within an intergenerational nursing workforce (n =* 1). The four generations of nursing professionals (X, Y, Baby Boomers, and Veterans) have different aptitudes, social and cultural setting, that coexist within the same work staff.

**Conclusion:**

This study establishes the legitimacy of the intergenerational differences as an important variable of social categorization. The findings have the potential to improve generational comprehension and promote a more cohesive culture in clinical practice settings, besides conserving the legacy of the four generations of nursing professionals contributing to outline the identity of the nurses through the conservation of social, cultural, and professional experiences.

## Resumen

**Objetivo:**

Describir las diferencias y similitudes generacionales existentes entre los profesionales de enfermería del siglo XX y XXI y cómo han influido en la evolución de la profesión.

**Métodos:**

Revisión integradora según la metodología de Whittemore y Knafl. Las palabras clave utilizadas para la búsqueda fueron: enfermeras, relaciones intergeneracionales, Veteranos, Baby Boom, generación X y Millennials.

**Resultados:**

El proceso de búsqueda electrónica dio como resultado un total de 10 documentos (8 artículos y dos tesis), todos ellos en el ámbito anglosajón (4 en Canadá, 5 en Estados Unidos y 1 en Australia). En los documentos recuperados se determinaron tres temas principales: *la fuerza de trabajo de enfermería intergeneracional (n =* 7), *el reclutamiento y la retención dentro de una fuerza de trabajo intergeneracional (n =* 2) y *la tutoría dentro de una fuerza laboral de enfermería intergeneraciona*l *(n =* 1). Las cuatro generaciones de profesionales de enfermería (X, Y, Baby Boomers y Veteranos) tienen diferentes aptitudes, entorno social y cultural, que conviven dentro de un mismo equipo de trabajo.

**Conclusión:**

Este estudio establece la legitimidad de las diferencias intergeneracionales como una importante variable de categorización social. Los hallazgos tienen el potencial de mejorar la comprensión generacional y fomentar una cultura más cohesiva en entornos de práctica clínica, además de conservar el legado de las cuatro generaciones de profesionales de enfermería lo que contribuye a perfilar las señas de identidad de las enfermeras mediante la conservación de experiencias sociales, culturales y profesionales.

## Resumo

**Objetivo:**

Descreva as diferenças e semelhanças geracionais entre os profissionais de enfermagem dos séculos XX e XXI e como elas influenciaram a evolução da profissão.

**Métodos:**

Revisão integrativa segundo a metodologia Whittemore e Knafl. As palavras-chave utilizadas para a busca foram: Enfermeiros, relações intergeracionais, Veteranos, Baby Boom, Geração X e Millennials.

**Resultados:**

O processo de busca eletrônica resultou em um total de 10 documentos (8 artigos e duas teses), todos da área anglo-saxônica (4 no Canadá, 5 nos Estados Unidos e 1 na Austrália). Três temas principais foram identificados nos documentos recuperados: a força de trabalho de enfermagem intergeracional (n = 7), recrutamento e retenção dentro de uma força de trabalho intergeracional (n = 2) e tutoria dentro de uma força de trabalho de enfermagem intergeracional (n = 1). As quatro gerações de profissionais de enfermagem (X, Y, Baby Boomers e Veteranos) possuem diferentes aptidões, meio social e cultural, que convivem dentro de uma mesma equipe de trabalho.

**Conclusão:**

Este estudo estabelece a legitimidade das diferenças intergeracionais como uma importante variável de categorização social. Os resultados têm potencial para melhorar a compreensão geracional e fomentar uma cultura mais coesa no cenário da prática clínica, além de preservar o legado das quatro gerações de profissionais de enfermagem, ajudando a moldar a identidade do enfermeiro por meio da preservação de experiências sociais, culturais e profissional.

## Introduction

This study focuses on the 20th and 21st centuries, given that it is the temporary space where more changes were experienced in the restructuring and organizing of nursing as profession. The last century, due to its proximity, facilitates access to the legacy that can be transmitted by professionals who, in spite of the time transpired, can contribute with their knowledge, perceptions, attitudes, and experiences in different health settings. It is necessary to consider that the different generations of nursing professionals (X generation, Y generation, Baby Boomers, and Veterans) can coexist within the same health staff.(1) Each generation is subject to the development processes of the course of human life; each experiences a unique historical context that shapes the development of said course of life. The diversity of characteristics, like age, gender, socioeconomic level or even ethnicity can cause differences not only within the very work staff but within a generation with respect to the following. Understanding and accepting these differences can contribute to diminishing generational conflict.(2, 3) Due to this, it is fundamental to know the generational diversity(4) and address the specific needs of each of the generations.(5) The different perspectives provided by multiple generations may be used advantageously to enhance efficiency(6) and the results of the health staff and promote the resolution of generational conflicts to construct effective work teams.(7,8) Likewise, an environment in which nurses are respected for their differences(9) is key to generate commitment and promote satisfaction in the workplace. This is why, by fostering relationships, effective communication,(10,11) commitment,(12,13) and compensation(14) among nursing professionals from distinct generations, a cohesive team will be set up that reflects the shared values of all team members.

Through the bibliography search for articles that address generational themes, we set the purpose of appraising and describing knowledge of the generational differences or similarities existing among nursing professionals from the 20th and 21st centuries and how these have influenced on the evolution of the profession. These generational differences,(15) within the corresponding social and historical context, are not only known, but are at the service of common goals, thus, become a resource for learning and change. This is the paradigm that gives added value, converting the difference into advantage. In short, it is the paradigm that will be adopted to carry out this work and its justification.

The term generation(16) is used to identify the set of people, within similar age groups, born during the same moment of history and culture.(17) Although there is no absolute beginning or end among the different generations, overall, these encompass 15 - 20 years. The years included in each generation vary among researchers, particularly for those years on the cusp of a generation.(18) A thin line exists between segmenting and stereotyping generations, which is why stereotyping should not be done (19-21) of the nursing professionals for belonging to a given generation. It must be considered each nursing professionals have their personality and experiences and individual characteristics of life also combine to create unique beings.(22)

The term generational cohort (23) refers to people born during the same overall time lapse who share key vital experiences, including historical events, public heroes, entertainment, hobbies, and early work experiences. It is theorized that these common life experiences create cohesion in perspectives and attitudes. Although knowledge and skills increase as people age, the basic characteristics, including values and behavioral norms established during their formative years persist. As a result, generational cohorts develop values and distinct workforce patterns.

The 20th and 21st centuries include the GI generation,(24) the Veteran generation, the Baby Boom generation, the X generation, the Y generation, the Z generation (they have not yet joined the health teams) and talk has begun about the Alpha generation, 100% digital.(25) This study focuses exclusively on four generations that coincided in the workplace: (26) the Veteran generation, the Baby Boom generation, the X generation, and the Y generation. The following is a brief description of these generations.

The *generation of Veterans* (also called traditionalists, the Silent or War generation) comprises the nursing professionals born between 1925 and 1945. The Veteran generation has contributed importantly not only to the social, political, and economic transformation in Spain and the rest of the world, but it has been a bulwark within the nursing profession; laying the foundations of the nursing profession as we know it currently. Many of these professionals are already retired. During this period, dramatic events have taken place in the world, like the Great Depression, the Second World War, along with the Civil War in Spain, which led this generation to great sacrifice, like struggling and dying at the service of their respective countries. In turn, this impacted on the way these people saw the world of work. They believe in employment for life and in hierarchies. They also value professional respect, the professional image of nursing, loyalty, and dedication.(27) Veterans have worked hard and believe that hard work will produce rewards.(28) Changes make them uncomfortable and they tend to favor command, direction control and leadership styles.(29, 30) Their principals values are law and order, respect for authority, duty, honor, devotion, and sacrifice.

The Baby Boomer generation encompasses those born between 1946 - 1964; it was called Baby Boom due to due to the increased birth rate observed during this period, they currently constitute two thirds of all nursing professionals. The Baby Boomer generation is the biggest group among nursing professionals. An important number of nurses from the Baby Boomer generation retired in 2010.(7) They are known for their strong work ethic. They enjoy direct traditional communication, like face-to-face meetings, but have also adapted to les personal modern communication methods that use technology.(31,32) Overall, grew up in a two-parent home with a mother at home, a father who was an authority figure(33) and prefer teamwork.(19) Baby Boomers want the world to know they have achieved something,(34, 35) equating work with self-esteem;(36) consequently, they can be motivated by public recognition and work advantages. They are pictured as addicted to work and live to work.^37^

The X generation, born between 1965 and 1980, as they mature, are quickly becoming one of the pillars of the organizations; their strength is ideal to solve problems of the clinical practice or issues related with the guarantee of quality. The X generation is significantly smaller than that of the Baby Boomers. They have been described as the latchkey children of parents with two careers. They are not too loyal to leaders(38) and institutions, seeing education as a necessary tool to survive in a competitive world. They are slow to commit and value both their personal and professional lives. They show more indicators of burnout(39,40) and are less inclined to participate in the exchange of knowledge.(41)

The Millennials generation, also known as the Y generation, the Net generation, or next generation is composed of nursing professionals born between 1981 and 2000; members of the Y generation and children of the Baby Boomers. By absolute numbers, this generation alone far exceeds the Baby Boom generation, driven in part by an increase in the immigrant population. The Y generation has grown in a multicultural and multiethnic world. Communication through technology is the cornerstone of this generation with mobile phones, text messages, and e-mail. They are experts in technology.(42) An expanding economy encouraged values, like optimism, trust, honesty, accomplishments,(43) career advancement,(44) la sociability and morality. They are self-sufficient(29) and value teamwork,(45,46) as well as tutoring(47-51) and feedback. Just like the Veterans, nursing professionals belonging to the Y generation expect rewards for hard work. The Millennials have an altruistic desire to help,(52) value the balance between work and life,(53) want to make decisions on their work schedules.(54) One of the most interesting characteristics of the Millennials is their expectation of having the capacity to contribute to decisions in their workplace, provoked by their active role in family decisions.(55)

## Methods

The search was conducted in the following databases: CINAHL, PubMed, ProQuest, EbscoHost, Science direct, Scopus, Web of Science, Wiley on-line library, Ovid. In addition, the search was extended to secondary references and to the manual search of journals. The keywords used for the search were: nurses, intergenerational relations, Veterans, Baby Boom, X generation, Millennials.

The selection criteria were descriptive, quantitative, or qualitative studies, and mixed-method studies, which describe the knowledge, perceptions, attitudes, and experiences of the four generations of nursing professionals in different health settings, including students and which additionally contain studies by nursing professionals from the point of view of the four generation cohorts (X generation, Y generation, Baby Boomers, and Veterans). The work included gray literature due to its important source of information that can be corroborated by experts on the field, besides being able to report on useful scientific findings that can reduce publication bias. Other inclusion criteria were peer-reviewed studies, without date-of-publication limit and in English.

The work excluded articles missing any of the four generations of nursing professionals, studies that did address the perspective of any of the four generations or studies whose samples were not constituted by nursing professionals, including students. It also excluded articles that did not describe in detail the knowledge and attitudes of the health professionals in relation with the four generations or that addressed other themes not defined in this study. The study also exclude d presentation formats, like books, text chapters, editorials, and comments or reviews**.**

To evaluate the quality of the studies included (quantitative and qualitative), a quality verification list was used**.**^56^ This verification list comprises nine questions, each of which has four subcategories. A total score is calculated of methodological quality, which varies from 9 (quite poor) to 36 (good) and quantitative and qualitative studies can be analyzed, including gray literature. The quality score of the studies included ranges between 33 and 36.

Four research questions were used: what is the current state of the literature with respect to the intergenerational nursing workforce and its influence on the evolution of this profession, what is the current state of the literature with respect to recruiting, retention and tutoring of nurses within an intergenerational workforce and their influence on the evolution of this profession, what is the potential for future research with respect to the nursing profession and its intergenerational work environment, do intergenerational studies exist on nursing professionals in Spain? The updated integrative review methodology described by Whittemore and Knafl(57) was used as guide for this review. An integrative review is a specific review method that summarizes past empirical or theoretical literature to provide more complete comprehension of a theme or phenomenon besides playing an important role in the evidence-based practice for nursing.(58)

## Results

**Figure 1:**
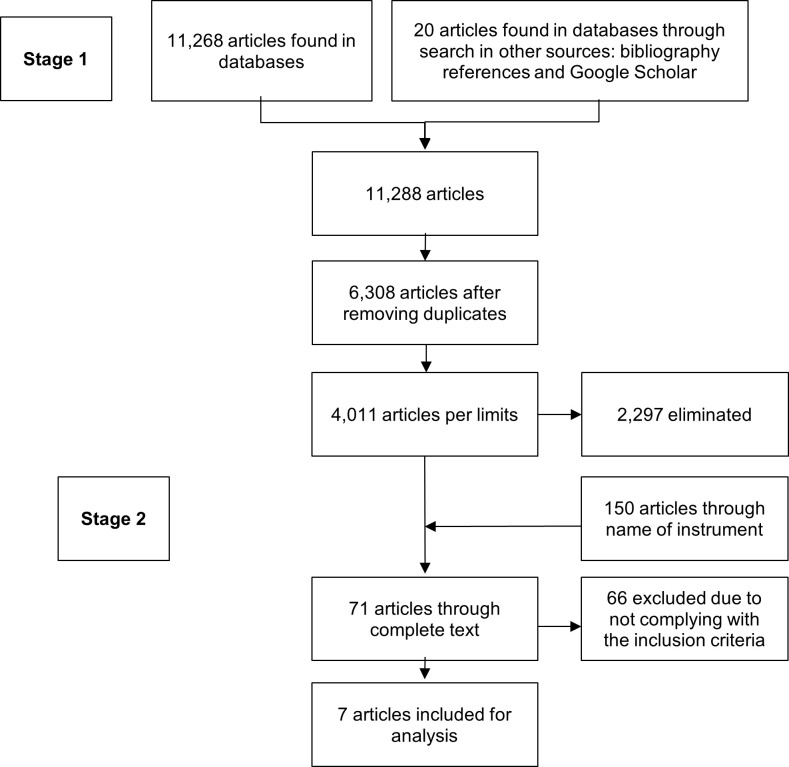
Heuristic and document selection process

The electronic search process yielded eight articles and two thesis works. Figure 1 summarizes the heuristic and selection process.

The work included 10 articles,(9,12,13,18,27,29,30,44,49,53); two from gray literature.(9,53) The articles included in this study all belong to the Anglo-Saxon setting**:** four from Canada(18, 30,44,49), one from Australia(29) and five from the United States(9,12,13,27,53). No study was found in the Spanish context containing the four generations of nursing professionals. Three themes were determined: *intergenerational nursing workforce* with seven articles;(9,12,13,18,27,29,30) *recruiting and retaining within an intergenerational workforce* with two articles;(44,53) *tutoring within an intergenerational nursing workforce* with one article.(49) Table 1 summarizes the research studies.

**Table 1:** Summary of research studies

Theme 1. The intergenerational nursing workforce
**Blythe *et al.,* Canada; 2008**(18)
*Objective:* to perform an exploratory analysis to determine if nurses of different ages had different attitudes toward their work.
*Design:* mixed methodology study.
*Data collection method: survey*s and focal groups.
*Sample: n* = 1,396. 96.2% were women.
*Quality score:* 33
Crowther and Kemp. Australia; 2009(29)
*Objective:* to determine how attitudes of nurses from rural mental health differ over generations.
*Design:* descriptive study.
*Data collection method:* surveys by year of birth.
*Sample: n* = 89.4 were Veterans, 52 Baby Boomers, 17 X generation, and 5 Y generation.
*Quality score:* 36
Hisel. USA; 2020(27)
*Objective:* to examine the level of job commitment among Veteran, Baby Boom, X Generation, and Millennial nurses.
*Design:* quantitative causal comparative design.
*Data collection method:* surveys through a social network platform to measure their level of job commitment.
*Sample: n* = 1,885. 92% were women.
*Quality score: 36*
**Hu *et al*., USA; 2004**(12)
*Objective:* to help management nurses to maximize departmental effectiveness by capitalizing the unique characteristics of the multigenerational nursing staff.
*Design:* descriptive design.
*Data collection method:* surveys.
*Sample: n* = 62; 90,3% were women.
*Quality score: 35*
MacDonnell and Buck- Fadyen. Canada; 2017(30)
*Objective:* to explore the critical influences that determine the meanings, practices, and impacts of nursing activism.
*Design:* qualitative exploratory study, comparative study of life story that uses a feminist lens.
*Data collection method:* interviews and focal groups.
*Sample: n* = 40. X generation = 8, Y generation = 9, Baby Boomers = 20, and Veterans = 3. 87.5% were women.
*Quality score:* 33
**Sullivan *et al.,* USA; 2013**(13)
*Objective:* to describe the job commitment of nursing professionals, identify generational predictors, present the implications for nursing managers, and suggest future research.
*Design:* descriptive study.
*Data collection method:* non-experimental surveys.
*Sample: n* = 747
*Quality score:* 36
Welcher. USA; 2011(9)
*Objective:* to explore generational conflicts related with four generations working together and the values, beliefs and attitudes of each generation in local hospitals in Georgia.
*Design:* qualitative phenomenological study using the Van Kaam method modified by Moustakas (1994)
*Data collection method:* interviews.
*Sample: n* = 20
*Quality score:* 35
Theme 2. Recruiting and retaining an intergenerational workforce
Steinkuehler. USA; 2009(53)
*Objective:* to review related literature and conduct an exploratory research on the organizational attraction of multigenerational nursing cohorts in the health industry.
*Design:* Descriptive correlation study.
*Data collection method:* surveys. Questionnaires that focus on a computer-generated random stratified sample of nurses.
*Sample: n* = 1100. 250 veterans, 250 Baby Boomers, 300 X generation, and 300 Y generation participants.
*Quality score:* 35
**Tourangeau *et al.,* Canada; 2015**(44)
*Objective:* to describe the characteristics of the work of nursing professors and determine if generational differences exist.
*Design:* descriptive study.
*Data collection method:* Phase I used focal groups. Phase II developed and used a survey.
*Sample: n* = 650
*Quality score:* 34
Theme 3. Tutoring within an intergenerational nursing workforce
**Earle *et al*., Canada; 2011**(49)
*Objective:* to discuss an integrative review of the literature.
*Design:* mixed-method study.
*Data collection method:* integrative review methodology de Whittemore and Knafl (2005).
*Sample: n* = 13,188. 18 articles
*Quality score:* 33

## Discussion

### Theme 1. The intergenerational nursing workforce

Older nurses, according to Blythe *et al*.,(39) were more committed with the workplace, had higher job satisfaction, and were less emotionally exhausted than the younger nurses. Hisel(27) also coincides on Veteran nurses as the generation most committed, followed by Baby Boom nurses, the X generation, and Millennials. Studies by Welcher(9) confirmed that the level of commitment emerged as the principal difference with respect to job habits and attitudes among the older nurses (Veteran generation and Baby Boom generation) and nurses from younger generations (X generation and Y generation). Nurses belonging to the Veteran and Baby Boomer generations tended to be more committed with work compared with the younger generations of nurses. However, authors, like Sullivan *et al.*,(13) also coincide in that Veterans were the generation most committed with work and in their study found that the X generation was the least committed. In addition, they conclude that the fact that the health staff is comprised of highly committed nurses contributes to providing quality care. Nevertheless, with the retirement of Veteran nurses and the upcoming retirement of the Baby Boom nurses, nurses from the X generation and the Millennials will become the dominant workforce in health care. Current medical care organizations must be prepared for this change toward a less committed nursing workforce.(27) In the study by Hu *et al.*,(12) almost half the Veterans and Baby Boomers consider computers as terrifying and complicated. The level of commitment and technology competence (9) were the principal differences in work habits between nurses from older and younger generations.

Veterans and Baby Boomers consider work and social life as one;(29) in addition, they value maintaining a single employer throughout their lives. While the Baby Boomers(9) accept long work shifts and take on overtime hours, the X generation values balance between work and family, so they work out of necessity. Regarding social activism,(30) the X and Y generations focused on social health determinants and social injustice for population groups; the Baby Boomers and Veterans identify activism as a central practice and a professional problem.

### Theme 2. Recruiting and retaining nurses within an intergenerational workforce

Within an intergenerational workforce, the X and Y generations(53) consider economic performance as more important than for the Veteran and Baby Boomer generations. Other studies(44) found that the Veteran generation selected health problems as a disincentive to stay employed. It must be kept in mind that at the time of the study the members in the Veteran generation were 66 years of age or older, which may explain the high rate of selection by this generation of this disincentive. To retain this older generation of nursing professors in academic settings, modifications could be made in their work to help them to comply effectively with their academic roles.

### Theme 3. Tutoring of nurses within an intergenerational workforce

Studies exist that highlight the importance(49) of tutoring within the work context in relation with recruiting and retaining nurses, considering tutoring the key support that younger nurses need to perform leadership roles. This study(49) also manifests that there is currently a disconnect between the educational values of students and the teaching staff, which increases awareness of the need to examine further these intergenerational differences. In short, taking advantage of the contribution of the skill set of each individual cohort and each generation of nursing professionals, more cohesive work teams can be formed.(28) By mutually supporting the distinct generations of nurses their contribution to patient care is maximized.( 35)

Limitations of the bibliography review include selection of sources, and lack of visibility of publications that are not indexed in databases. To avoid publication bias, gray literature was used. Note that representation has not been found of studies conducted in Europe and even in Spain that include the four generations of nursing professionals studied. It must be kept in mind that the sociopolitical circumstances of each country are different and the social and cultural development of the late 20th century has not been simultaneous in every region of the planet. This may be a line of study for future research. Only one study exists from the gender perspective. Due to the lack of reference regarding the gender of participants in the studies or samples that do not turn out statistically significant in terms of the generational cohorts, it is not possible to analyze the study from the gender perspective.

## Conclusion

Since the late 20^th^ century and early 21^st^ century care quality has been deteriorating as a consequence of the shortage^59^ of nursing professionals globally. The shortage of nurses is the product of the combination of an aging workforce, high rotation of nursing staff and the lack of capacity to attract and retain these professionals. Hence, those responsible for health services must know how to promote work commitment in young generations, bearing in mind that different authors have concluded that the X generation and Millennials are the least committed, given that Veteran and Baby Boom nurses have retired or are close to retiring. Only international studies exist that include the four generations of nursing professionals (X, Y, Baby Boomers; Veterans) in the Anglo-Saxon setting, finding none in the Spanish context, which may be proposed for future research. Similarly, future research may be proposed on intergenerational studies from the gender perspective. The findings have the potential to improve generational comprehension and foster a more-cohesive culture in clinical practice settings, besides conserving the legacy of the four generations of nursing professionals, contributing to outline the identity of the nurses through the conservation of social, cultural, and professional experiences.
